# Interplay of strain and race/ethnicity in the innate immune response to *M*. *tuberculosis*

**DOI:** 10.1371/journal.pone.0195392

**Published:** 2018-05-22

**Authors:** P. Nahid, L. G. Jarlsberg, M. Kato-Maeda, M. R. Segal, D. H. Osmond, S. Gagneux, K. Dobos, M. Gold, P. C. Hopewell, D. M. Lewinsohn

**Affiliations:** 1 Division of Pulmonary and Critical Care Medicine, San Francisco General Hospital, University of California, San Francisco, United States of America; 2 Department of Epidemiology & Biostatistics, University of California, San Francisco, United States of America; 3 Swiss Tropical and Public Health Institute, Department of Medical Parasitology and Infection Biology, University of Basel, Basel, Switzerland; 4 Colorado State University, Department of Microbiology, Immunology & Pathology, Fort Collins, CO, United States of America; 5 Department of Research, Veterans Affairs Portland Health Care Center, Portland, Oregon, United States of America; 6 Department of Pulmonary and Critical Care Medicine, Oregon Health and Sciences University, Portland, Oregon; 7 Department of Research, Veterans Affairs Portland Health Care Center, Portland, Oregon, United States of America; Institut de Pharmacologie et de Biologie Structurale, FRANCE

## Abstract

**Background:**

The roles of host and pathogen factors in determining innate immune responses to *M*. *tuberculosis* are not fully understood. In this study, we examined host macrophage immune responses of 3 race/ethnic groups to 3 genetically and geographically diverse *M*. *tuberculosis* lineages.

**Methods:**

Monocyte-derived macrophages from healthy Filipinos, Chinese and non-Hispanic White study participants (approximately 45 individuals/group) were challenged with *M*. *tuberculosis* whole cell lysates of clinical strains Beijing HN878 (lineage 2), Manila T31 (lineage 1), CDC1551 (lineage 4), the reference strain H37Rv (lineage 4), as well as with Toll-like receptor 2 agonist lipoteichoic acid (TLR2/LTA) and TLR4 agonist lipopolysaccharide (TLR4/LPS). Following overnight incubation, multiplex assays for nine cytokines: IL-1β, IL-2, IL-6, IL-8, IL-10, IL-12p70, IFNγ, TNFα, and GM-CSF, were batch applied to supernatants.

**Results:**

Filipino macrophages produced less IL-1, IL-6, and more IL-8, compared to macrophages from Chinese and Whites. Race/ethnicity had only subtle effects or no impact on the levels of IL-10, IL-12p70, TNFα and GM-CSF. In response to the Toll-like receptor 2 agonist lipoteichoic acid (TLR2/LTA), Filipino macrophages again had lower IL-1 and IL-6 responses and a higher IL-8 response, compared to Chinese and Whites. The TLR2/LTA-stimulated Filipino macrophages also produced lower amounts of IL-10, TNFα and GM-CSF. Race/ethnicity had no impact on IL-12p70 levels released in response to TLR2/LTA. The responses to TLR4 agonist lipopolysaccharide (TLR4/LPS) were similar to the TLR2/LTA responses, for IL-1, IL-6, IL-8, and IL-10. However, TLR4/LPS triggered the release of less IL-12p70 from Filipino macrophages, and less TNFα from White macrophages.

**Conclusions:**

Both host race/ethnicity and pathogen strain influence the innate immune response. Such variation may have implications for the development of new tools across TB therapeutics, immunodiagnostics and vaccines.

## Introduction

Genotyping of *Mycobacterium tuberculosis* (*M*. *tuberculosis*) has shown that lineages of the organism have predilections for certain geographic distributions and dominate certain geographic areas [1–4]. Members of the East Asian lineage (that includes the Beijing family) are predominant in East Asia, members of the Indo-Oceanic lineage are predominant in the Philippines and around the rim of the Indian Ocean [5], and members of the *M*. *africanum* West Africa I and II are largely restricted to West Africa [1, 6]. This global distribution of strains may not be random or based solely upon variabilities in virulence, but may be partly determined by the genetics, epigenetics and other factors of the host and the microbe, both of which are under natural selection forces.

Successful containment of infection with *M*. *tuberculosis* is dependent on innate immune responses, as these responses play a central role in the acquisition of the adaptive T cell response, in granuloma formation, and ultimately in the containment of intracellular growth of *M*. *tuberculosis* [7]. The cells responsible for the containment of intracellular infection, dendritic cells and alveolar macrophages, are also the preferred intracellular host of *M*. *tuberculosis*. Central to the function of these cells is responsiveness to bacterially derived patterns, that are recognized by Toll-like receptors (TLR). There is evidence that TLRs play an important role in the host immune response to *M*. *tuberculosis* [7]. Alveolar macrophages are the first responders to inhalation of tubercle bacilli and represent an important component of the innate response to *M*. *tuberculosis* infection. Exposure of macrophages to *M*. *tuberculosis* results in the secretion of cytokines, including TNFα, GM-CSF, IL-1α, IL-1β, IL-6, IL-8, IL-10, and IL-12p70, that play key roles in granuloma formation [8–10]. Two members of the mammalian TLR family, TLR2 and possibly TLR4, have been found to recognize mycobacterial products and to mediate macrophage activation and the ensuing production of particular cytokines [7, 11]. The discovery of TLR polymorphisms that are associated with race/ethnicity and response to particular pathogens, raises the distinct possibility that there are ethnicity-specific differences in TLR2 and/or TLR4, resulting in differential innate responses to *M*. *tuberculosis* strains [12–16]. Such immunologic differences in innate response are of interest given the molecular epidemiologic studies demonstrating differences in the transmissibility and virulence of *M*. *tuberculosis* based on strain [17] [18–20]. Differential activation through TLRs could have a significant impact on the host response to infection with *M*. *tuberculosis*, and ultimately to disease progression.

Our study explores the interaction of host and pathogen factors on the innate immune response. Based on the molecular epidemiology of TB in San Francisco, which includes a sizable population of Asian patients [17, 21], we selected 3 race/ethnic groups, Filipinos, Chinese, and non-Hispanic Whites for inclusion in our study. From these healthy donors, macrophages were isolated and treated with lysates from 4 *M*. *tuberculosis* strains, comprising 3 predominant lineages identified in San Francisco (East Asian [Beijing HN878], Indo-Oceanic [Manila T31] and EuroAmerican [CDC1551]), and one laboratory reference strain (H37Rv), or with agonists of TLR2 (lipoteichoic acid LTA) or TLR4 (lipopolysaccharide LPS). A total of 9 cytokines were measured. This study adds to the growing body of evidence that differential innate immune responses contribute to ethnicity-associated differences in TB susceptibility.

## Methods

### Ethics statement

The “Innate Immunity to TB” (IITB) study protocol was reviewed and approved by the committee on human research at the University of California, San Francisco (CHR# H45279-29459). Written informed consent was obtained from all study participants.

### Study design and participants

Healthy volunteers, aged 18 to 55, self-identifying as Chinese, Filipino, or non-Hispanic White were invited to participate in the study. Recruitment was through bulletin board postings at the Schools of Dentistry, Medicine, Nursing, Pharmacy, Physical Therapy of the University of California, San Francisco, and at the undergraduate and graduate schools of San Francisco State University. Between 2009 and 2011, all consenting volunteers meeting inclusion and having no exclusion criteria were consecutively enrolled into IITB. Recruitment continued until 50 participants per race/ethnic group were enrolled. All volunteers completed a standardized questionnaire reviewing demographics, health history as well as information on potential risk factors for TB. All volunteers reported the race/ethnicity of their biological parents and grandparents, and only those identifying both generations as Chinese, Filipino or non-Hispanic White were eligible for participation in the study. Those reporting a history of active TB, evidence of prior TB on chest radiograph, vaccination with Mycobacterium bovis bacillus Calmette-Guérin (BCG) vaccine in the past 15 years, co-morbid conditions including diabetes, renal disease, cancer, hepatitis, HIV/AIDS, or other moderate or high risk characteristics associated with TB, such as homelessness or history of incarceration, significant alcohol use (greater than 2 drinks per day), tuberculin skin testing within past 6 months, or treatment with any immunomodulating agents such as corticosteroids, were not eligible to participate. All participants had IFN-γ release assay testing, specifically Quantiferon-Gold® (QFT), for latent TB infection at the time of study enrollment.

### Mycobacterial strains, TLR agonists

Whole cell lysates of Beijing HN878, Manila T31, CDC1551, and H37Rv, were obtained from gamma-irradiated cells as described previously [22]. The total protein content from each whole cell lysate batch was quantified by bicinchoninic acid assay (ThermoFisher Scientific, Walthham, MA), and samples were portioned into individual 10 mg vials and stored at -80c until use. Certificates of Analysis and Product Information Sheets for whole cell lysates are available through the National Institute of Allergy and Infectious Diseases funded BEI Resources (http://www.beiresources.org/; catalog numbers: NR-14820, NR-14821, NR-14824, NR-36496).

### Isolation, culture and ex vivo stimulation of Monocyte Derived Macrophages (MDMs)

For each study participant, phlebotomy was performed to fill four BD Vacutainer® CPT™ Cell Preparation Tube with Sodium Citrate (Becton, Dickinson and Company, Franklin Lakes, NJ, No. 362761). Whole blood was processed according to manufacturer guidance for the separation of mononuclear cells. Monocytes were prepared from freshly drawn blood samples through negative selection using the Miltenyi Biotec Monocyte Isolation Kit II, which provides an indirect magnetic labeling approach for the isolation of untouched monocytes from human peripheral blood mononuclear cells (PBMCs). Monocytes were counted and resuspended at 5x10^5^/ml in 10% vol/vol human serum (HuS), and then plated at 500,000/well in ultralow adherence plates, without stimulation, and incubated at 37°C with CO_2_ for 72 hours to obtain enrichment of monocyte-derived macrophages (MDMs) through adherence. Upon harvesting of MDMs, viability was assessed by absence of uptake of trypan blue, and purity (ranging 96–98% MDMs) was determined by fluorescence cell sorter (FACS). We further assessed viability on the basis of HLA-DR and CD11c, and T cell contamination on the basis of CD3. MDMs from each study participant, were incubated at 1x10^5^ for 18 hours with 10 ug / ml whole cell lysate of *M*. *tuberculosis* strains, and to agonists of TLR2 (10 ug / ml Lipoteichoic acid (LTA) from S. aureus, Sigma, L 2515) and TLR4 (50 ng/ml lipopolysaccharide (LPS) from E. coli Sigma, L-2880); and culture medium (negative control). Following overnight incubation, supernatants were collected and frozen at -80°C and batch tested using MSD Human ProInflammatory 9-Plex Tissue Culture Kit K15007B-1 (measuring GM-CSF, IFN-γ, IL-1β, IL-10, IL-12p70, IL-2, IL-6, IL-8, TNF-α) run on a Meso Scale Diagnostics MESO QuickPlex SQ 120 instrument.

### Statistics and analysis

Categorical demographic factors were analyzed using chi squared tests; age was compared by race using the Student’s t-test. Geometric mean cytokine responses were compared in response to 6 stimulants (4 *M*. *tuberculosis* strains: H37Rv “reference strain,” CDC1551 “EuroAmerican,” HN878 “East Asian,” and T31 “Indo-Oceanic;” and 2 stimulants on TLR pathways: LTA and LPS) and by race (White, Chinese, and Filipino). For analyses where stimulant was held constant and responses were compared by race, linear regression was used, adjusting for independent factors age and gender. For analyses where race was held constant and participant responses to each stimulant were compared, a general estimating equation (GEE) was added to the linear regression to adjust for responses clustered by participant, in addition to adjusting for age and gender [23]. Multivariable linear regression models were used to assess interplay between race and strain as predictors of cytokine response. P values were adjusted for multiple testing error using the false discovery rate (FDR) method, and significance was defined at the *p* < 0.20 level [24].

Cytokines were measured in picograms (pg), logged to approximate a normal distribution for analysis, and presented as geometric means with 95% confidence intervals. The upper limit of detection (ULOD) was 10,000 pg for all cytokines; values above the ULOD were set to 10,000 pg. The lower limits of detection (LLOD) were unique for each cytokine; values below the LLOD were left unchanged. All statistical analyses were performed using SAS version 9.4 (SAS Institute, Cary, NC, USA).

## Results

### Study population

The study population included 137 healthy donors with mean age 30±8 (range 19–55) years of age. Most were female (n = 99, 72%), and 39% were foreign-born (n = 54). A positive QFT test was uncommon (8%) and more likely to occur among the foreign-born (RR 15.4, 95%CI 2–116). Self-identified race was divided approximately evenly: non-Hispanic White (n = 46), Chinese (n = 47), or Filipino (n = 44). Filipinos were on average older, and Whites were less likely to be foreign-born (Table 1).

**Table 1:** Demographic characteristics of the study population.

					*p* value	
	White	Chinese	Filipino	White vs. Chinese	White vs. Filipino	Chinese vs. Filipino
**n (%)**	46 (34)	47 (34)	44 (32)			
**Mean age±SD** ** (Range)**	28.3±6.8 (22–53)	28.7±8.3 (19–55)	32.9±7.1 (22–49)	0.815	0.002	0.005
**Female, n (%)**	30 (65)	34 (73)	35 (80)	0.459	0.132	0.423
**Foreign birth, n (%)**	5 (11)	22 (47)	27 (61)	<0.001	<0.001	0.165
**QFT positive, n (%)**	1 (2)	4 (9)	6 (14)	0.201	0.071	0.436

### Cytokine levels

We prepared monocyte-derived macrophages (MDMs) from healthy study participants (n = 137), and incubated the cells with *M*. *tuberculosis* lysates. The concentration of cytokines released in response to the bacterial lysates were measured using 2 different platforms (Luminex Assay and Meso Scale Discovery immunoassay (MSD); Meso Scale Discovery, Gaithersburg, Maryland) according to manufacturer instructions. Since the cytokine levels and trends were identical for these 2 platforms, we describe our data generated with the MSD kit. We measured a total of 9 cytokines: IL-1β, IL-2, IL-6, IL-8, IL-10, IL-12p70, IFNγ, TNFα, and GM-CSF. As the study was designed to assess macrophage stimulation, the T cell cytokines IFN-γ and IL-2 were included as controls. IFNγ and IL-2, at the lower limit of detection was noted, possibly due T cell contamination.

### Cytokine response to M. tuberculosis strain by race/ethnicity

**Fig 1:**
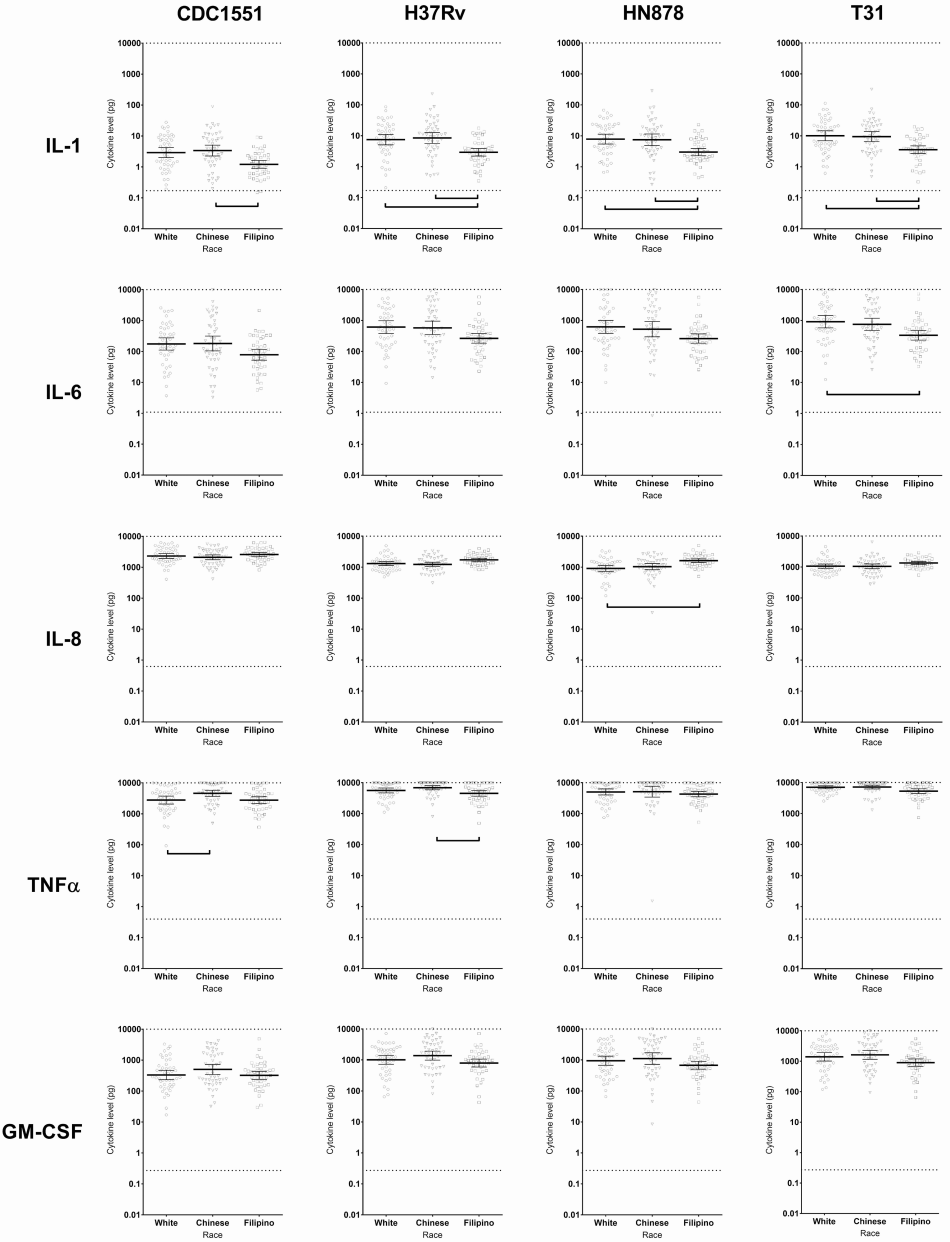
Cytokine response of macrophages to *M*. *tuberculosis* strains by race/ethnicity. Cytokines released by macrophages derived from healthy individuals of three ethnicities in response to *M*. *tuberculosis* lysates from four strains. Comparisons are adjusted for age and gender. Brackets indicate FDR-adjusted *p* < 0.20.

We observed similar patterns across all 4 *M*. *tuberculosis* strains for IL-1b, IL-6 and IL-8 (Fig 1 and S1 Table). Filipino MDMs produced less IL-1 and IL-6, but more IL-8, compared to MDMs from Whites and Chinese. We observed subtle differences across *M*. *tuberculosis* strains for TNFα, and GM-CSF. For TNFα, Filipino and White MDMs released less compared to Chinese MDMs, in response to CDC1551. Filipino MDMs released less of this cytokine than Chinese cells, in response to H37Rv, and Filipino MDMs released less TNFα than both Chinese and White MDMs in response to T31. There was no difference by race/ethnicity in the amounts of TNFα released in response to HN878. Only strain T31 triggered the release of different GM-CSF amounts across race/ethnic groups: Filipino MDMs released less of this cytokine, compared to Chinese MDMs (Fig 1 and S1 Table). There were no race/ethnic differences in IL-10 and IL-12p70 responses to any of the *M*. *tuberculosis* strains (S1 Table).

### Cytokine response to TLR agonists by race/ethnicity

MDMs were incubated for 18 hours with the TLR2 agonist LTA or the TLR4 agonist LPS, and cytokine responses were measured.

**Fig 2:**
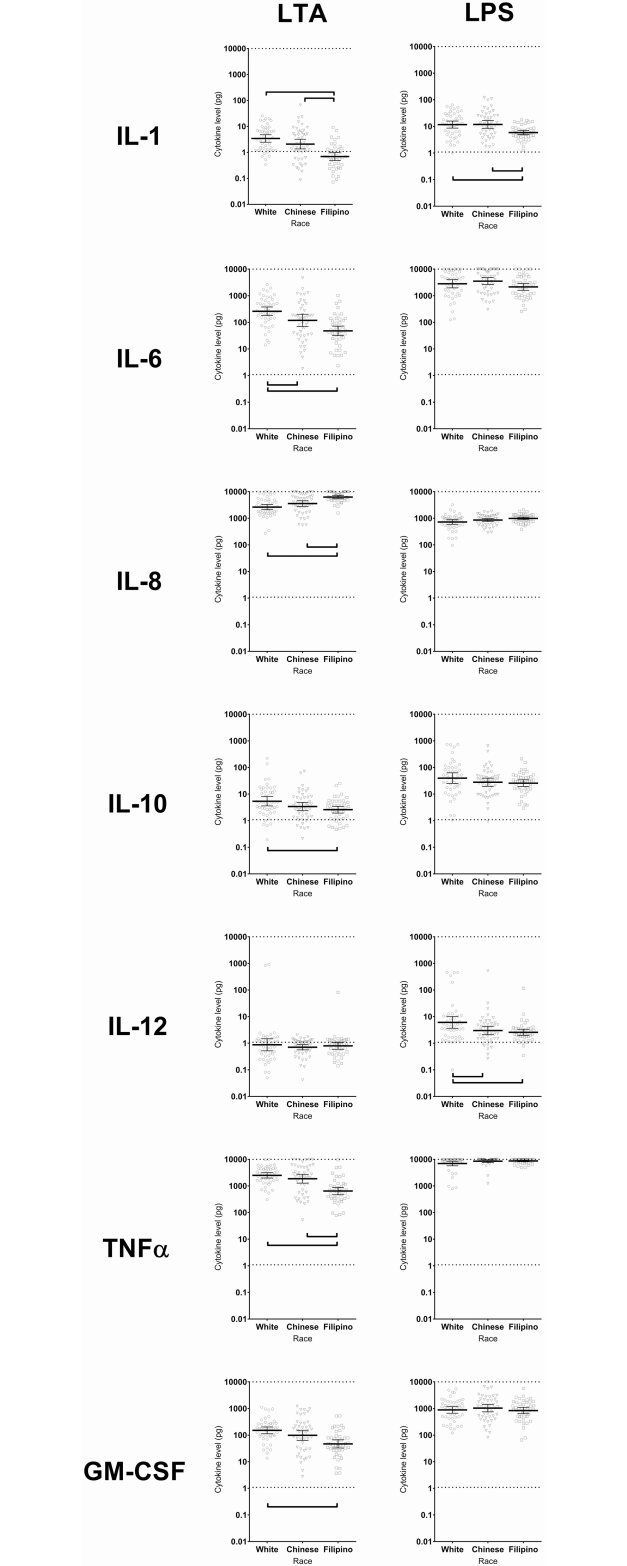
Cytokine response of macrophages to Toll-like receptor agonists by race/ethnicity. Cytokines released by macrophages derived from healthy individuals of three ethnicities in response to TLR2 agonist lipoteichoic acid (LTA) and TLR4 agonist lipopolysaccharide (LPS). Comparisons are adjusted for age and gender. Brackets indicate FDR-adjusted *p* < 0.20.

In response to TLR2/LTA, Filipino MDMs released lower amounts of IL-1, IL-6, IL-10, TNFα and GM-CSF, but higher amounts of IL-8, compared to Whites and Chinese (Fig 2 and S2 Table). There were no differences by race/ethnicity in the IL-12p70 response to TLR2/LTA (S2 Table).

In response to TLR4/LPS, Filipino MDMs released lower amounts of IL-1, IL-6, IL-10, IL-12p70 but higher amounts of IL-8, compared to Whites and Chinese, while White MDMs released lower amounts of TNFα, compared to Chinese (Fig 2 and S2 Table). There were no differences across race/ethnicity groups in the GM-CSF responses to TLR4/LPS (S2 Table).

### Cytokine response by M. tuberculosis strain

We explored the impact of *M*. *tuberculosis* strain on cytokine responses within each race/ethnicity (S1 Fig and S3 Table). Strain CDC1551 elicited lower levels of IL-1, IL-6, IL-10, TNFα and GM-CSF, and higher levels of IL-8, compared to all other strains, regardless of race/ethnicity. Strain T31 triggered the release of more IL-12p70 compared to the other strains, regardless of race/ethnicity. These data indicate that the clinical *M*. *tuberculosis* strains CDC1551 and T31 may have different immunogenic profiles, compared to *M*. *tuberculosis* strains H37Rv and HN878, recognizing that cellular lysates are a crude proxy of infection.

### Cytokine response by both race/ethnicity and strain

Using a multivariable linear regression model predicting response of macrophages to *M*. *tuberculosis* lysates and race/ethnicity, adjusted for age and gender, Filipino ethnicity was an independent predictor of lower IL-1 and IL-6 response, and of higher IL-8 response compared to Whites (Table 2). CDC1551 strain was an independent predictor of lower IL-1, IL-6, IL-10, TNFα, and GM-CSF response, and of higher IL-8 response compared to the reference strain H37RV. In addition, T31 (Manila strain) was an independent predictor of lower IL-8 activity, and higher activity in IL-1, IL-6, IL-10, IL-12p70, TNFα, and GM-CSF compared to H37RV. HN878 strain was independently predictive of lower IL-8, TNFα, and GM-CSF response compared to H37RV. When multiple testing correction is applied no significant interactions emerged.

**Table 2:** Seven multivariable linear regression models predicting response of macrophages to *M*. *tuberculosis* lysates and race/ethnicity, adjusted for age and gender. General estimating equation included to adjust for values clustered by participant. P values adjusted for multiple testing using the false discovery rate (FDR) method.

	IL-1		IL-6		IL-8		IL-10		IL-12p70		TNFα		GM-CSF	
	Estimate (95%CI)	p value	Estimate (95%CI)	p value	Estimate (95%CI)	p value	Estimate (95%CI)	p value	Estimate (95%CI)	p value	Estimate (95%CI)	p value	Estimate (95%CI)	p value
**Race**														
White	Ref	1.00	Ref	1.00	Ref	1.00	Ref	1.00	Ref	1.00	Ref	1.00	Ref	1.00
Chinese	0.06 (-0.41, 0.53)	.890	-0.05 (-0.67, 0.57)	.929	-0.02 (-0.21, 0.18)	.929	0.02 (-0.43, 0.47)	.949	-0.08 (-0.48, 0.32)	.868	0.19 (-0.05, 0.43)	.224	0.27 (-0.15, 0.70)	.332
Filipino	-0.76 (-1.2, -0.36)	.001	-0.70 (-1.2, -0.17)	.023	0.22 (0.04, 0.39)	.032	-0.15 (-0.57, 0.27)	.686	-0.20 (-0.66, 0.25)	.577	-0.11 (-0.37, 0.14)	.577	-0.12 (-0.52, 0.27)	.756
**Strain**														
H37RV	Ref	1.00	Ref	1.00	Ref	1.00	Ref	1.00	Ref	1.00	Ref	1.00	Ref	1.00
CDC1551	-0.92 (-1.0, -0.79)	< .001	-1.2 (-1.3, -1.1)	< .001	0.51 (0.45, 0.57)	< .001	-1.0 (-1.1, -0.93)	< .001	-0.03 (-0.20, 0.14)	.868	-0.53 (-0.64, -0.43)	< .001	-1.0 (-1.1, -0.88)	< .001
HN878	-0.02 (-0.14, 0.10)	.868	-0.03 (-0.15, 0.08)	.787	-0.20 (-0.30, -0.10)	< .001	0.06 (-0.08, 0.19)	.577	0.18 (-0.05, 0.41)	.224	-0.16 (-0.29, —0.03)	.035	-0.15 (-.26, -0.04)	.013
T31	0.20 (0.11, 0.30)	< .001	0.30 (0.21, 0.39)	< .001	-0.20 (-0.24, -0.15)	< .001	0.15 (0.10, 0.21)	< .001	0.17 (0.10, 0.24)	< .001	0.13 (0.07, 0.19)	< .001	0.20 (0.14, 0.27)	< .001
**Covariates**														
Age	-0.04 (-0.06, -0.02)	.001	-0.04 (-0.07, -0.01)	.025	0.02 (0.01, 0.03)	.002	0.00 (-0.02, 0.02)	.988	-0.04 (-0.06, -0.01)	.010	-0.01 (-0.03, 0.00)	.290	-0.03 (-0.05, -0.01)	.032
Male	0.07 (-0.36, 0.50)	.868	0.04 (-0.50, 0.58)	.929	-0.03 (-0.21, 0.15)	.868	-0.39 (-0.80, 0.02)	.121	0.32 (-0.04, 0.67)	.151	0.04 (-0.19, 0.27)	.868	0.06 (-0.33, 0.45)	.868

## Discussion

In this study of the response of macrophages derived from healthy individuals of 3 race/ethnic groups to *M*. *tuberculosis* lysates from 4 phylogeographic strains of the organism, we found that both host race/ethnicity and pathogen strain influence the innate immune response.

We found that macrophages from Filipinos produced less IL-1, IL-6, and more IL-8, compared to macrophages from Chinese and Whites participants. In response to the Toll-like receptor 2 agonist LTA, Filipino macrophages again had lower IL-1 and IL-6 responses and a higher IL-8 response, compared with Chinese and Whites. The TLR2/LTA-stimulated Filipino macrophages also produced lower amounts of IL-10, TNFα and GM-CSF, and the TLR4/LPS triggered the release of less IL-12p70 from Filipino macrophages, and less TNFα from White macrophages. These data would suggest that the induction of, IL-6, TNF-α, GM-CSF could be associated with TLR2 ligation. Unexpectedly, the induction of IL-8 appeared to be distinct from that of IL-6. In this regard, induction of both IL-6 and IL-8 via TLR-2 are both MyD88 dependent [25]. The differential response of IL-8 would imply either that other receptors might play a selective role in IL-8 induction. For example, the mannose receptor has been previously demonstrated to enable TLR-2 dependent IL-8 production. Similarly, Dectin1 and CD36 could modulate TLR responsiveness. Interestingly, *M*. *tuberculosis* can activate leukocytes via the mannose receptor [26], although this possibility has not been formally tested.

We also examined the effect of pathogen strain using lysates prepared from 4 *M*. *tuberculosis* strains: clinical strains Beijing HN878 (lineage 2), Manila T31 (lineage 1) and CDC1551 (lineage 4), and reference strain H37Rv (lineage 4. We found that CDC1551 elicited a markedly different macrophage response, for most of the cytokines, eliciting less IL-1, IL-6, IL-10, TNFα, and GM-CSF, and more IL-8, compared with other *M*. *tuberculosis* strains. These data would appear to parallel those seen for specific TLR-2 stimulation. Our data, then, would suggest that both differential responses to each strain, as well as those seen among the race/ethnic groups are driven by TLR-2 signalling, and would not support the original hypothesis that each strain would be “tuned” to its concordant, geographically-aligned host.

TB susceptibility and disease outcome vary greatly among individuals, and it is well established that at least some of this variability is attributable to host genetics [27–31]. Genetic loci that may influence clinical TB phenotypes have been identified [31–35]. Most of these genes encode immune response proteins [31, 36–38]. Here, responsiveness and/or production of IFN-γ has been frequently observed as strongly associated with vulnerability to mycobacterial infection. With regard to TLRs it has been argued that TLR signaling specifically has “yet to provide convincing evidence of a major contribution of common variants of human TLRs, IL-1Rs, or their adaptors to host defense” [39]. Nonetheless, there a number of examples where specific TLRs or molecules related to TLR signaling can be associated with vulnerability to infection with Mtb [15, 16], or to altered responsiveness to immunization with BCG [40]. Our data with regard to TLR2 responsiveness strongly supports genetic variation in TLR2 signaling.

The impact of ethnicity on TB susceptibility was revealed over a quarter century ago [41]. One of the first studies to dissect the relative influence of ethnicity and *M*. *tuberculosis* strain on TB disease phenotype showed that ethnicity is a powerful predictor of disease phenotype, independently of pathogen strain [42]. However, few studies have addressed the basis of these ethnic differences at the cellular and molecular levels. At the cellular level, one report has suggested that the ability of *M*. *tuberculosis* to grow in macrophages varies according to the ethnicity of the macrophage donor [43]. At the molecular level, a TLR2 polymorphism appears to be associated with TB susceptibility in Asians, but not in Caucasians [44], and inflammatory mediator levels are associated with ethnicity in pulmonary TB patients of African and Eurasian ancestry [45]. These ethnic differences are associated with ethnic variation in host vitamin D binding protein *DBP* phenotype, in accord with the demonstrated roles of the DBP protein in macrophage activation and neutrophil chemotaxis [46, 47]. Here, we show that macrophages derived from healthy Filipino donors had markedly different innate immune responses to *M*. *tuberculosis* lysate, to TLR2 stimulation with LTA, and to TLR4 stimulation with LPS, as compared with macrophages from non-Hispanic Whites and Chinese. It will be of interest to expand these studies to other ethnicities, including Africans, African-Americans and Hispanics.

At present, information regarding ethnic differences in TLR SNPs is limited. For example, one ethnic-specific difference is SNP TLR1 rs5743618 (changes T to G at bp 1805 and changes I to S at aa 602) [48]. This SNP regulates IL6 response to PAM3 stimulation, presumably via the ligation of the TLR1/2 heterodimer. The high responding variant is dominant in Asians (99% T) and Blacks (75%) while the low responding variant dominates in Whites and (75%). The entire TLR1/2/6 locus is an evolutionary hot spot under positive selection [48].

Many epidemiological and experimental studies have shown that *M*. *tuberculosis* strains produce different disease phenotypes and immune responses [20, 49–51]. This clinical phenotype diversity across *M*. *tuberculosis* strains is likely attributable to differing gene expression [51–53], metabolic profiles [54], cell wall lipids [55] and growth rates in macrophages [56]. We show here that clinical strain CDC1551 (lineage 4 modern) triggers the release of less IL-1, IL-6, IL-10, TNFα, and GM-CSF, and more IL-8, compared with clinical strains Beijing HN878 (lineage 2 modern), Manila T31 (lineage 1 ancient) and reference strain H37Rv (lineage 4 modern). Our results are in accord with those of Subbian et al, who reported that rabbits infected with CDC1551 produced less lung TNFα mRNA compared to rabbits infected with HN878, although, contrary to our data, they found that CDC1551 also triggered the production of less IL-8 mRNA [57]. As IL-8 is associated with neutrophilc inflammation, it was postulated that this was one reason that CDC1115 induced less cavitation. Manca et al. compared the innate immune responses elicited by strains CDC1551, HN878 and H37Rv, with results that are not congruent with our study. Mice infected with CDC1551 produced more IL-6 and IL-10 mRNA than HN878 and H37Rv, and more TNFα mRNA than HN878 [58, 59], and human monocytes infected with CDC1551 produced more IL-10 and TNFα mRNA than cells infected with HN878 [60]. These discordant results likely reflect different experimental systems, and it is notable that the earlier studies relied on small sample sizes.

The molecular bases for these innate immune response differences across host ethnicity and *M*. *tuberculosis* pathogen lineages are largely unknown. However, comparative genomics, and ongoing efforts to understand the unusual biology of these bacteria, including unbiased systems biology approaches [51, 61], are expected to yield insights. Furthermore, our work would support a detailed evaluation of TLR-2 dependent signaling across each of these species.

Our study has limitations, principally that our experimental system did not include a model in which macrophages were infected with live *M*. *tuberculosis*. We recognize that the use of cellular lysates represents a proxy of infection useful for comparisons rather than definitive determination of immunogenicity of strains. Also, whereas macrophages for all assays were enumerated at 50,000 cells/well, we did not enumerate circulating monocytes in volunteers nor evaluate for cell surface expression, which may have affected induced cytokine differences. Additionally, due to the multiplexed nature of assays and resource limitations, we were unable to conduct diluted assays, resulting in a censored upper limit of detection. Finally, ethnicity was determined based on self-report rather than genetic testing. Whereas, it has been shown that self-definitions of ethnicity are highly accurate reflection of true ethnic identity in Chinese and Whites, in particular, there is the potential for misclassification of ethnicity [62]. Our study also has a number of strengths. First, the immunologic responses to *M*. *tuberculosis* were evaluated using cells from healthy individuals and focused on innate immunity, thus reducing the influence of adaptive immunity, history of TB exposure, as well as environmental and socioeconomic factors. Second, our controlled experimental design allowed us to examine the interaction of host and pathogen genetics. Third, our study population of 137 healthy macrophage donors lends considerable robustness to our results.

In sum, our data show that that both host race/ethnicity and pathogen factors influence the innate immune response and such variation has implications for the development of new tools across TB therapeutics, immunodiagnostics and vaccines [3, 45, 63].

## Supporting information

S1 Fig Cytokine response of macrophages by *M*. *tuberculosis* strain. Cytokines released by macrophages derived from healthy individuals of three ethnicities in response to *M*. *tuberculosis* lysates from four strains. Comparisons are adjusted for age and gender. Brackets indicate FDR-adjusted *p* < 0.20.
(DOCX)

S1 Table Cytokine response (geometric mean (GM) levels) of macrophages to lysates of four *M*. *tuberculosis* strains, by race/ethnicity, and adjusted for age and gender. P values adjusted for multiple testing using the false discovery rate (FDR) method.
(DOCX)

S2 Table Cytokine response (geometric mean (GM) levels) of macrophages to TLR2 agonist LTA and TLR4 agonist LPS, by race/ethnicity, and adjusted for age and gender. P values adjusted for multiple testing using the false discovery rate (FDR) method.
(DOCX)

S3 Table Cytokine response (geometric mean (GM) levels) of macrophages to *M*. *tuberculosis* lysates, by strain, and adjusted for age and gender. General estimating equation is included to adjust for cytokine values clustered by participant. P values adjusted for multiple testing using the false discovery rate (FDR) method.
(DOCX)
