# Supplementary material for: Interplay of strain and race/ethnicity in the innate immune response to M. tuberculosis
Source: PLoS One. 2018 May 22;13(5):e0195392. doi: 10.1371/journal.pone.0195392 (PMC5963792; doi:10.1371/journal.pone.0195392)
Supplement: S1 Fig — Cytokines released by macrophages derived from healthy individuals of three ethnicities in response to M. tuberculosis lysates from four strains. Comparisons are adjusted for age and gender. Brackets indicate FDR-adjusted p < 0.20. (DOCX) [file pone.0195392.s001.docx]

**Supporting Information PONE-D-17-38141**

**Nahid et al., Interplay of strain and race/ethnicity in the innate immune response to *M. tuberculosis***

**S1 Fig**. **Cytokine response of macrophages by *M. tuberculosis* strain.** Cytokines released by macrophages derived from healthy individuals of three ethnicities in response to *M. tuberculosis* lysates from four strains. Comparisons are adjusted for age and gender. Brackets indicate FDR-adjusted *p* < 0.20.

***

***
