# Supplementary material for: Interplay of strain and race/ethnicity in the innate immune response to M. tuberculosis
Source: PLoS One. 2018 May 22;13(5):e0195392. doi: 10.1371/journal.pone.0195392 (PMC5963792; doi:10.1371/journal.pone.0195392)
Supplement: S1 Table — P values adjusted for multiple testing using the false discovery rate (FDR) method. (DOCX) [file pone.0195392.s002.docx]

**Supporting Information PONE-D-17-38141**

**Nahid et al., Interplay of strain and race/ethnicity in the innate immune response to *M. tuberculosis***

**S1 Table. Cytokine response (geometric mean (GM) levels) of macrophages to lysates of four *M. tuberculosis* strains, by race/ethnicity, and adjusted for age and gender. P values adjusted for multiple testing using the false discovery rate (FDR) method.**

| **Cytokine** | **CDC1551** | | | **H37Rv** | | | **HN878** | | | **T31** | | |
| --- | --- | --- | --- | --- | --- | --- | --- | --- | --- | --- | --- | --- |
| Race/Ethnicity | **GM (95%CI)** | ***p*** | | **GM (95%CI)** | ***p*** | | **GM (95%CI)** | ***p*** | | **GM (95%CI)** | ***p*** | |
| **IL-1** |  |  |  |  |  |  |  |  |  |  |  |  |
| White | 2.91 (2.00-4.25) | Ref |  | 7.52 (5.09-11.1) | Ref |  | 7.84 (5.43-11.3) | Ref |  | 10.1 (6.96-14.6) | Ref |  |
| Chinese | 3.38 (2.26-5.06) | .731 | Ref | 8.48 (5.66-12.7) | .759 | Ref | 7.51 (4.90-11.5) | .931 | Ref | 9.47 (6.51-13.8) | .892 | Ref |
| Filipino | 1.21 (0.91-1.62) | .069 | .016 | 2.93 (2.21-3.89) | .038 | .016 | 3.00 (2.29-3.94) | .026 | .030 | 3.58 (2.70-4.75) | .016 | .016 |
| **IL-6** |  |  |  |  |  |  |  |  |  |  |  |  |
| White | 175 (111-276) | Ref |  | 611 (381-979) | Ref |  | 620 (381-1009) | Ref |  | 915 (577-1449) | Ref |  |
| Chinese | 181 (104-315) | .931 | Ref | 577 (351-950) | .931 | Ref | 522 (296-921) | .789 | Ref | 750 (475-1183) | .759 | Ref |
| Filipino | 78.4 (52.4-117) | .198 | .168 | 264 (184-379) | .182 | .198 | 261 (184-369) | .158 | .265 | 332 (233-473) | .045 | .139 |
| **IL-8** |  |  |  |  |  |  |  |  |  |  |  |  |
| White | 2313 (1935-2765) | Ref |  | 1306 (1125-1515) | Ref |  | 909 (733-1128) | Ref |  | 1070 (917-1249) | Ref |  |
| Chinese | 2103 (1757-2517) | .708 | Ref | 1230 (1049-1443) | .757 | Ref | 1038 (826-1305) | .668 | Ref | 1052 (882-1256) | .892 | Ref |
| Filipino | 2591 (2235-3002) | .860 | .496 | 1724 (1526-1946) | .197 | .056 | 1618 (1415-1851) | .016 | .067 | 1351 (1214-1502) | .359 | .245 |
| **IL-10** |  |  |  |  |  |  |  |  |  |  |  |  |
| White | 4.87 (3.21-7.39) | Ref |  | 13.0 (9.41-17.8) | Ref |  | 20.9 (13.8-31.5) | Ref |  | 17.7 (12.8-24.6) | Ref |  |
| Chinese | 5.91 (4.04-8.65) | .759 | Ref | 16.8 (12.1-23.4) | .546 | Ref | 14.8 (9.87-22.3) | .381 | Ref | 19.2 (13.7-26.8) | .892 | Ref |
| Filipino | 5.21 (3.82-7.09) | .978 | .759 | 15.3 (11.7-20.1) | .789 | .759 | 12.9 (9.53-17.3) | .205 | .763 | 15.5 (11.8-20.4) | .691 | .546 |
| **IL-12p70** |  |  |  |  |  |  |  |  |  |  |  |  |
| White | 0.93 (0.55-1.57) | Ref |  | 0.80 (0.60-1.07) | Ref |  | 1.48 (0.74-2.98) | Ref |  | 0.98 (0.76-1.28) | Ref |  |
| Chinese | 0.75 (0.55-1.01) | .759 | Ref | 0.88 (0.62-1.25) | .759 | Ref | 0.99 (0.64-1.54) | .634 | Ref | 1.06 (0.80-1.41) | .769 | Ref |
| Filipino | 0.64 (0.46-0.89) | .759 | .996 | 0.69 (0.48-0.99) | .883 | .892 | 0.58 (0.39-0.86) | .279 | .759 | 0.77 (0.56-1.04) | .832 | .664 |
| **TNFα** |  |  |  |  |  |  |  |  |  |  |  |  |
| White | 2784 (2075-3733) | Ref |  | 5620 (4686-6741) | Ref |  | 5028 (4017-6292) | Ref |  | 7042 (6295-7878) | Ref |  |
| Chinese | 4606 (3681-5763) | .041 | Ref | 6906 (5913-8065) | .234 | Ref | 5121 (3430-7645) | .931 | Ref | 7149 (6203-8238) | .892 | Ref |
| Filipino | 2775 (2160-3566) | .892 | .070 | 4540 (3686-5591) | .576 | .045 | 4290 (3504-5252) | .759 | .757 | 5253 (4403-6266) | .111 | .067 |
| **GM-CSF** |  |  |  |  |  |  |  |  |  |  |  |  |
| White | 332 (234-469) | Ref |  | 1013 (727-1411) | Ref |  | 947 (674-1332) | Ref |  | 1397 (1019-1914) | Ref |  |
| Chinese | 502 (344-732) | .234 | Ref | 1384 (992-1931) | .307 | Ref | 1110 (723-1703) | .757 | Ref | 1628 (1163-2278) | .716 | Ref |
| Filipino | 323 (239-436) | .892 | .355 | 796 (593-1068) | .883 | .211 | 675 (505-902) | .728 | .334 | 903 (681-1197) | .486 | .168 |
