# Supplementary material for: Interplay of strain and race/ethnicity in the innate immune response to M. tuberculosis
Source: PLoS One. 2018 May 22;13(5):e0195392. doi: 10.1371/journal.pone.0195392 (PMC5963792; doi:10.1371/journal.pone.0195392)
Supplement: S2 Table — P values adjusted for multiple testing using the false discovery rate (FDR) method. (DOCX) [file pone.0195392.s003.docx]

**Supporting Information PONE-D-17-38141**

**Nahid et al., Interplay of strain and race/ethnicity in the innate immune response to *M. tuberculosis***

**S2 Table. Cytokine response (geometric mean (GM) levels) of macrophages to TLR2 agonist LTA and TLR4 agonist LPS, by race/ethnicity, and adjusted for age and gender. P values adjusted for multiple testing using the false discovery rate (FDR) method.**

| **Cytokine** | **LTA** | | | **LPS** | | |
| --- | --- | --- | --- | --- | --- | --- |
| Race/Ethnicity | **GM (95%CI)** | ***p*** | | **GM (95%CI)** | ***p*** | |
| **IL-1** |  |  |  |  |  |  |
| White | 3.45 (2.48-4.79) | Ref |  | 11.7 (8.63-15.9) | Ref |  |
| Chinese | 2.09 (1.36-3.22) | .087 | Ref | 11.8 (8.47-16.5) | .934 | Ref |
| Filipino | 0.69 (0.49-0.99) | <.001 | .005 | 5.88 (4.91-7.05) | .014 | .011 |
| **IL-6** |  |  |  |  |  |  |
| White | 260 (179-378) | Ref |  | 2814 (1971-4016) | Ref |  |
| Chinese | 118 (69.6-201) | .034 | Ref | 3541 (2629-4770) | .413 | Ref |
| Filipino | 47.8 (31.6-72.3) | <.001 | .076 | 2153 (1607-2884) | .408 | .081 |
| **IL-8** |  |  |  |  |  |  |
| White | 2637 (2093-3324) | Ref |  | 725 (594-884) | Ref |  |
| Chinese | 3584 (2804-4580) | .076 | Ref | 858 (751-981) | .202 | Ref |
| Filipino | 6276 (5553-7093) | <.001 | .014 | 987 (885-1101) | .081 | .622 |
| **IL-10** |  |  |  |  |  |  |
| White | 5.37 (3.59-8.04) | Ref |  | 39.8 (25.2-62.9) | Ref |  |
| Chinese | 3.41 (2.38-4.88) | .081 | Ref | 27.9 (19.6-39.8) | .201 | Ref |
| Filipino | 2.59 (1.94-3.48) | .011 | .385 | 25.7 (19.3-34.2) | .087 | .672 |
| **IL-12p70** |  |  |  |  |  |  |
| White | 0.88 (0.52-1.48) | Ref |  | 6.08 (3.63-10.2) | Ref |  |
| Chinese | 0.71 (0.56-0.89) | .519 | Ref | 3.01 (2.09-4.32) | .033 | Ref |
| Filipino | 0.80 (0.59-1.07) | .934 | .575 | 2.58 (1.98-3.34) | .032 | .928 |
| **TNFα** |  |  |  |  |  |  |
| White | 2499 (1990-3139) | Ref |  | 6973 (5713-8512) | Ref |  |
| Chinese | 1871 (1278-2738) | .314 | Ref | 8550 (7624-9590) | .081 | Ref |
| Filipino | 646 (469-892) | <.001 | <.001 | 8674 (8147-9234) | .120 | .928 |
| **GM-CSF** |  |  |  |  |  |  |
| White | 151 (112-203) | Ref |  | 886 (666-1178) | Ref |  |
| Chinese | 99.5 (63.6-153) | .169 | Ref | 1035 (753-1422) | .519 | Ref |
| Filipino | 46.8 (32.9-66.5) | .001 | .068 | 838 (635-1107) | .944 | .575 |
