# Supplementary material for: Interplay of strain and race/ethnicity in the innate immune response to M. tuberculosis
Source: PLoS One. 2018 May 22;13(5):e0195392. doi: 10.1371/journal.pone.0195392 (PMC5963792; doi:10.1371/journal.pone.0195392)
Supplement: S3 Table — General estimating equation is included to adjust for cytokine values clustered by participant. P values adjusted for multiple testing using the false discovery rate (FDR) method. (DOCX) [file pone.0195392.s004.docx]

**Supporting Information PONE-D-17-38141**

**Nahid et al., Interplay of strain and race/ethnicity in the innate immune response to *M. tuberculosis***

**S3 Table. Cytokine response (geometric mean (GM) levels) of macrophages to *M. tuberculosis* lysates, by strain, and adjusted for age and gender. General estimating equation is included to adjust for cytokine values clustered by participant. P values adjusted for multiple testing using the false discovery rate (FDR) method.**

| **Cytokine** | **White** | | | | **Chinese** | | | | **Filipino** | | | |
| --- | --- | --- | --- | --- | --- | --- | --- | --- | --- | --- | --- | --- |
| Race | **GM (95%CI)** | ***p*** | | | **GM (95%CI)** | ***p*** | | | **GM (95%CI)** | ***p*** | | |
| **IL-1** |  |  |  |  |  |  |  |  |  |  |  |  |
| CDC1551 | 2.91 (2.00-4.25) | Ref |  |  | 3.38 (2.26-5.06) | Ref |  |  | 1.21 (0.91-1.62) | Ref |  |  |
| H37Rv | 7.52 (5.09-11.1) | <.001 | Ref |  | 8.48 (5.66-12.7) | <.001 | Ref |  | 2.93 (2.21-3.89) | <.001 | Ref |  |
| HN878 | 7.84 (5.43-11.3) | <.001 | .777 | Ref | 7.51 (4.90-11.5) | <.001 | .162 | Ref | 3.00 (2.29-3.94) | <.001 | .805 | Ref |
| T31 | 10.1 (6.96-14.6) | <.001 | .004 | .050 | 9.47 (6.51-13.8) | <.001 | .095 | .003 | 3.58 (2.70-4.75) | <.001 | .029 | .078 |
| **IL-6** |  |  |  |  |  |  |  |  |  |  |  |  |
| CDC1551 | 175 (111-276) | Ref |  |  | 181 (104-315) | Ref |  |  | 78.4 (52.4-117) | Ref |  |  |
| H37Rv | 611 (381-979) | <.001 | Ref |  | 577 (351-950) | <.001 | Ref |  | 264 (184-379) | <.001 | Ref |  |
| HN878 | 620 (381-1009) | <.001 | .917 | Ref | 522 (296-921) | <.001 | .413 | Ref | 261 (184-369) | <.001 | .762 | Ref |
| T31 | 915 (577-1449) | <.001 | <.001 | .004 | 750 (475-1183) | <.001 | <.001 | .001 | 332 (233-473) | <.001 | <.001 | <.001 |
| **IL-8** |  |  |  |  |  |  |  |  |  |  |  |  |
| CDC1551 | 2313 (1935-2765) | Ref |  |  | 2103 (1757-2517) | Ref |  |  | 2591 (2235-3002) | Ref |  |  |
| H37Rv | 1306 (1125-1515) | <.001 | Ref |  | 1230 (1049-1443) | <.001 | Ref |  | 1724 (1526-1946) | <.001 | Ref |  |
| HN878 | 909 (733-1128) | <.001 | <.001 | Ref | 1038 (826-1305) | <.001 | .109 | Ref | 1618 (1415-1851) | <.001 | .214 | Ref |
| T31 | 1070 (917-1249) | <.001 | <.001 | .104 | 1052 (882-1256) | <.001 | <.001 | .917 | 1351 (1214-1502) | <.001 | <.001 | <.001 |
| **IL-10** |  |  |  |  |  |  |  |  |  |  |  |  |
| CDC1551 | 4.87 (3.21-7.39) | Ref |  |  | 5.91 (4.04-8.65) | Ref |  |  | 5.21 (3.82-7.09) | Ref |  |  |
| H37Rv | 13.0 (9.41-17.8) | <.001 | Ref |  | 16.8 (12.1-23.4) | <.001 | Ref |  | 15.3 (11.7-20.1) | <.001 | Ref |  |
| HN878 | 20.9 (13.8-31.5) | <.001 | .002 | Ref | 14.8 (9.87-22.3) | <.001 | .278 | Ref | 12.9 (9.53-17.3) | <.001 | <.001 | Ref |
| T31 | 17.7 (12.8-24.6) | <.001 | <.001 | .325 | 19.2 (13.7-26.8) | <.001 | <.001 | .011 | 15.5 (11.8-20.4) | <.001 | .801 | <.001 |
| **IL-12p70** |  |  |  |  |  |  |  |  |  |  |  |  |
| CDC1551 | 0.93 (0.55-1.57) | Ref |  |  | 0.75 (0.55-1.01) | Ref |  |  | 0.64 (0.46-0.89) | Ref |  |  |
| H37Rv | 0.80 (0.60-1.07) | .568 | Ref |  | 0.88 (0.62-1.25) | .069 | Ref |  | 0.69 (0.48-0.99) | .354 | Ref |  |
| HN878 | 1.48 (0.74-2.98) | .305 | .049 | Ref | 0.99 (0.64-1.54) | .101 | .556 | Ref | 0.58 (0.39-0.86) | .182 | .018 | Ref |
| T31 | 0.98 (0.76-1.28) | .805 | <.001 | .216 | 1.06 (0.80-1.41) | <.001 | .012 | .556 | 0.77 (0.56-1.04) | .003 | .143 | .002 |
| **TNFα** |  |  |  |  |  |  |  |  |  |  |  |  |
| CDC1551 | 2784 (2075-3733) | Ref |  |  | 4606 (3681-5763) | Ref |  |  | 2775 (2160-3566) | Ref |  |  |
| H37Rv | 5620 (4686-6741) | <.001 | Ref |  | 6906 (5913-8065) | <.001 | Ref |  | 4540 (3686-5591) | <.001 | Ref |  |
| HN878 | 5028 (4017-6292) | <.001 | .273 | Ref | 5121 (3430-7645) | .560 | .093 | Ref | 4290 (3504-5252) | <.001 | .070 | Ref |
| T31 | 7042 (6295-7878) | <.001 | .002 | .001 | 7149 (6203-8238) | <.001 | .457 | .061 | 5253 (4403-6266) | <.001 | <.001 | <.001 |
| **GM-CSF** |  |  |  |  |  |  |  |  |  |  |  |  |
| CDC1551 | 332 (234-469) | Ref |  |  | 502 (344-732) | Ref |  |  | 323 (239-436) | Ref |  |  |
| H37Rv | 1013 (727-1411) | <.001 | Ref |  | 1384 (992-1931) | <.001 | Ref |  | 796 (593-1068) | <.001 | Ref |  |
| HN878 | 947 (674-1332) | <.001 | .615 | Ref | 1110 (723-1703) | <.001 | .027 | Ref | 675 (505-902) | <.001 | <.001 | Ref |
| T31 | 1397 (1019-1914) | <.001 | <.001 | <.001 | 1628 (1163-2278) | <.001 | <.001 | <.001 | 903 (681-1197) | <.001 | .007 | <.001 |
